# Right median nerve electrical stimulation for acute traumatic coma (the Asia Coma Electrical Stimulation trial): study protocol for a randomised controlled trial

**DOI:** 10.1186/s13063-017-2045-x

**Published:** 2017-07-10

**Authors:** Xiang Wu, Chao Zhang, Junfeng Feng, Qing Mao, Guoyi Gao, Jiyao Jiang

**Affiliations:** Department of Neurosurgery, Renji Hospital, School of Medicine, Shanghai Jiaotong University, Shanghai, China

**Keywords:** Traumatic brain injury, Comatose patient, Right median nerve electrical stimulation

## Abstract

**Background:**

Traumatic brain injury (TBI) has become the most common cause of death and disability in persons between 15 and 30 years of age, and about 10–15% of patients affected by TBI will end up in a coma. Coma caused by TBI presents a significant challenge to neuroscientists. Right median nerve electrical stimulation has been reported as a simple, inexpensive, non-invasive technique to speed recovery and improve outcomes for traumatic comatose patients.

**Methods/design:**

This multicentre, prospective, randomised (1:1) controlled trial aims to demonstrate the efficacy and safety of electrical right median nerve stimulation (RMNS) in both accelerating emergence from coma and promoting long-term outcomes. This trial aims to enrol 380 TBI comatose patients to partake in either an electrical stimulation group or a non-stimulation group. Patients assigned to the stimulation group will receive RMNS in addition to standard treatment at an amplitude of 15–20 mA with a pulse width of 300 μs at 40 Hz ON for 20 s and OFF for 40 s. The electrical treatment will last for 8 h per day for 2 weeks. The primary endpoint will be the percentage of patients regaining consciousness 6 months after injury. The secondary endpoints will be Extended Glasgow Outcome Scale, Coma Recovery Scale-Revised and Disability Rating Scale scores at 28 days, 3 months and 6 months after injury; Glasgow Coma Scale, Glasgow Coma Scale Motor Part and Full Outline of Unresponsiveness scale scores on day 1 and day 7 after enrolment and 28 days, 3 months and 6 months after injury; duration of unconsciousness and mechanical ventilation; length of intensive care unit and hospital stays; and incidence of adverse events.

**Discussion:**

Right median nerve electrical stimulation has been used as a safe, inexpensive, non-invasive therapy for neuroresuscitation of coma patients for more than two decades, yet no trial has robustly proven the efficacy and safety of this treatment. The Asia Coma Electrical Stimulation (ACES) trial has the following novel features compared with other major RMNS trials: (1) the ACES trial is an Asian multicentre randomised controlled trial; (2) RMNS therapy starts at an early stage 7–14 days after the injury; and (3) various assessment scales are used to evaluate the condition of patients. We hope the ACES trial will lead to optimal use of right median nerve electrical treatment.

**Trial registration:**

ClinicalTrials.gov, NCT02645578. Registered on 23 December 2015.

**Electronic supplementary material:**

The online version of this article (doi:10.1186/s13063-017-2045-x) contains supplementary material, which is available to authorized users.

## Background

The incidence of traumatic brain injury (TBI), which is the most common cause of death and disability in persons between 15 and 30 years of age, has increased as a result of heavy traffic [1, 2]. Approximately 10–15% of patients with severe TBI end up in a coma or vegetative state [1, 3, 4]. It is now believed that coma is a self-limiting state that typically evolves within 2–4 weeks into a vegetative state (VS), minimally conscious state (MCS), or conscious state (CS) [5]. A VS is a condition of wakeful unconsciousness in which patients can open their eyes spontaneously but cannot understand, communicate or behave purposefully. An MCS is a condition of severely altered consciousness characterised by minimal but definite behavioural evidence of self-awareness or environmental awareness. The examiner may elicit clear evidence of volitional behaviour on one examination, but fail to do so during a subsequent examination conducted hours or even minutes later [6, 7]. Coma and its unfavourable successions, VS and MCS, have become a heavy burden for families and society.

Neuroscientists are working on how to speed recovery and improve the functional outcomes and prognosis of these patients. Treatments including pharmacological interventions, right median nerve stimulation (RMNS), sensory stimulation, dorsal column stimulation, transcranial magnetic stimulation, deep brain stimulation and hyperbaric oxygen therapy have all been used to better achieve rehabilitation goals [3, 8–12]. No treatment has been proven robustly to alter the pace of recovery or improve the neurological outcomes of comatose patients following TBI.

As a simple, inexpensive, non-invasive technique, RMNS for coma arousal has a history of more than two decades. The application of electrical current to the extremities to treat central nervous system injury was first introduced in 1972 at the University of Virginia [13]. Radio-linked electrodes were surgically implanted on the bilateral femoral and sciatic nerves of a paraplegic patient to produce a semblance of walking using an external switch. Unexpectedly, in the mid-1980s researchers at Duke Biomedical Engineering not only noted a significant improvement in motor responses to electrical pulses in the stimulated arm of a quadriplegic subject, but also discovered a crossover effect of improvement in the strength of the proximal muscles of the unstimulated arm [14, 15]. This observation of intracerebral transfer led to the development of median nerve electrical stimulation for coma arousal. The first article about median nerve electrical stimulation for acute coma was published in 1999 [16]. Since then, RMNS has drawn increasing attention from many intensivists, rehabilitationists and clinical researchers [3, 5, 14, 17–23].

Among the articles published on RMNS, three randomised trials give some clues about the efficacy of this treatment. In the first randomised, double-blind study, a group of six comatose patients with TBI were randomised to receive RMNS treatment or sham stimulation. The RMNS group recovered more quickly, with a shorter intensive care unit (ICU) stay and improved Glasgow Coma Scale (GCS) and Glasgow Outcome Scale (GOS) scores 1 month after the injury [16]. In a double-blind randomised controlled trial with six RMNS-treated patients and four controls, the RMNS group emerged from coma 2 days earlier and scored higher in the Functional Independence Measure/Functional Assessment Measure (FIM/FAM) 3 months post injury [19]. In a third double-blind randomised controlled trial conducted by Lei and colleagues, 437 comatose patients with severe TBI were enrolled 2 weeks after their injury and assigned to the RMNS group or the control group according to their date of birth. The RMNS-treated patients had a more rapid increase in mean GCS, a significantly higher proportion of them regained consciousness and a lower proportion ended in VS. For those patients who regained consciousness, the FIM score was higher among the RMNS group [24].

Non-randomised controlled trial studies and reviews, although not as convincing, also indicate that RMNS may play a role in the emergence from coma following severe brain injury. For example, Liu and colleagues used RMNS on six comatose patients for 3 months. Four patients regained consciousness within 35 days, and brain perfusion increased in all six cases assessed by single-photon emission computed tomography (SPECT) scans after stimulation [20]. A review by Cooper and colleagues concluded that RMNS is a promising therapy for the neuroresuscitation of comatose patients, and time in the ICU may be shortened and the quality of the final outcome enhanced when the stimulation is applied early in the coma [18].

The trials selected had limitations due to the small number of cases analysed in most studies, inappropriate methods of randomisation in some cases, the diversity in coma length and coma severity, the heterogeneity of outcome measures, the different timing of intervention and follow-up and the lack of multicentre studies. We designed the ACES trial with an adequate sample size and a standardised protocol to obtain convincing evidence about the efficacy and safety of RMNS in both accelerating emergence from coma and promoting long-term outcomes.

## Methods/design

### Overview

A Standard Protocol Items: Recommendations for Interventional Trials (SPIRIT) checklist is available online for this manuscript (Additional file 1).

**Fig. 1:**
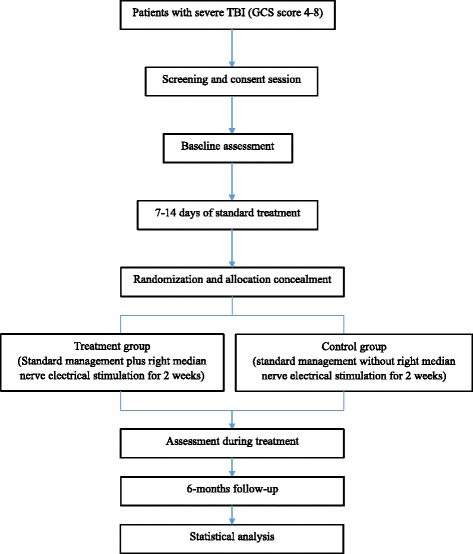
Overview of the flow of participants through the trial

**Fig. 2:**
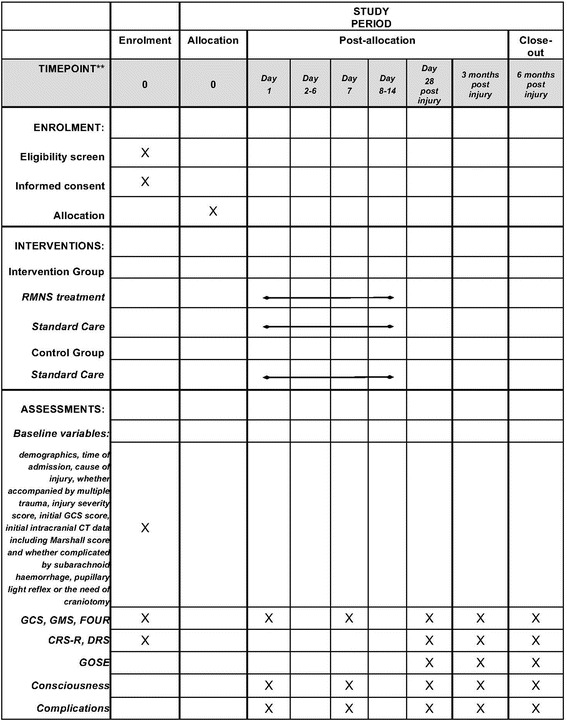
Schedule of enrolment, interventions and assessments. *GCS* Glasgow Coma Scale, *CT* computed tomography, *GMS* Glasgow Coma Scale Motor Part, *FOUR* Full Outline of Unresponsiveness (scale), *CRS-R* Coma Recovery Scale-Revised, *DRS* Disability Rating Scale, *GOSE* Extended Glasgow Outcome Scale

The Asia Coma Electrical Stimulation (ACES) trial is a prospective, Asian multicentre randomised controlled trial designed to examine the efficacy and safety of 2-week long right median nerve electrical stimulation in patients suffering from acute traumatic coma. Patients enrolled in the trial are randomly assigned to the stimulation group or the non-stimulation group. The primary endpoint, 6 months after injury, is the level of consciousness: VS, MCS, or CS (Fig. 1). Its design and the final report will follow the Consolidated Standards of Reporting Trials (CONSORT) statement as well as its extension to non-pharmacological interventions. The trial schedule is shown in Fig. 2.

### Study settings and population

A total of 380 patients will be recruited from 16 large, experienced, specialised neurosurgical centres across Asia: (1) Shanghai Renji Hospital; (2) Guangzhou General Hospital of Guangzhou Military Region; (3) the People’s Hospital of Shaoxin, Zhejiang; (4) the 98 Hospital of the People’s Liberation Army; (5) the Second People’s Hospital of Nanning, Guangxi; (6) the Central People’s Hospital of Tengzhou, Shandong; (7) Harrison International Peace Hospital of Hengshui, Hebei; (8) the 421 Hospital of the People’s Liberation Army; (9) Jiangning Hospital of Nanjing, Jiangsu; (10) the First People’s Hospital of Yulin, Guangxi; (11) the First People’s Hospital of Huaian, Jiangsu; (12) the People’s Hospital of Hunan; (13) the People’s Hospital of Pingyang, Zhejiang; (14) the Second People’s Hospital of Tianshui, Gansu; (15) the Fifth People’s Hospital of Zhengzhou University; (16) General Hospital of Beijing Military Region.

### Inclusion and exclusion criteria for the trial

Patients who meet the following criteria will be deemed eligible for the trial: (1) admitted to the hospital due to closed TBI 7 to 14 days previously; (2) GCS score of 4–8 or GMS below 5 on admission; and (3) aged 18–65 years old.

Those who meet the following criteria will be excluded from the trial: (1) vital signs not stable; (2) admitted to hospital due to penetrating cranial injury; (3) a confirmed history of epilepsy before enrolment or during the time of hospitalisation; (4) severe cardiac arrhythmia or pacemaker implanted; (5) pregnancy; and (6) no consent form.

### Ethics issues

The study protocol and consent forms have been approved by the Ethics Committee of Renji Hospital (NO: Renji Lunshen [2016] 001(2)) and the local institutional review boards of each participating site (Additional file 2). The trial is registered on ClinicalTrials.gov with ID number NCT02645578 and will be performed in accordance with the Declaration of Helsinki and the guidelines of Good Clinical Practice.

Theoretically, all patients should sign an informed consent form in person before enrolment. Due to the nature of TBI, the patients will not be physically or mentally capable of giving consent. A legal representative will be approached to give assent for participation in the trial after explanation by an investigator. The legal representative will usually be a family member or the closest relative who can make the decision on behalf of the patient. If no legal representative is available in due time, an independent staff member from the local institutional review boards who is not involved in the trial will be asked for study approval.

### Randomisation and allocation concealment

Participants at each site will be randomly assigned in a ratio of 1:1, stratified by study centre, to the stimulation group or the non-stimulation group, using a block-randomisation scheme. The whole randomisation procedure will be carried out by independent statisticians who are not involved in the determination of the final result. The trial secretary will place the computer-generated randomisation sequence and treatment assignment together into opaque envelopes and seal and mail these envelopes to each participating centre. After each patient is stabilised, assessed and identified as possibly meeting the criteria, a study physician at each local centre will do the group assignment by opening sequentially numbered envelopes.

### Treatment protocol

After being admitted to the hospital, all patients will be assessed and receive standard treatment (including surgery, if necessary) in the neurology ICU in accordance with the current guidelines [25]. Those who meet the criteria and whose representative has signed an informed consent form will be enrolled in the trial and be randomly assigned to the stimulation or non-stimulation group according to the procedure mentioned above.

The patients in the stimulation group will receive RMNS in addition to standard treatment. The electrical treatment will be delivered via a pair of lubricated 1-inch square rubber surface electrodes pasted 1 inch apart on the volar aspect of the right distal forearm over the median nerve. An electrical neuromuscular stimulator (Verity Medical Ltd., Braishfield, UK) will supply trains of asymmetric biphasic pluses at an amplitude of 15–20 mA (as tolerated) with a pulse width of 300 μs at 40 Hz ON for 20 s and OFF for 40 s, which has been proven to be well tolerated without causing pain or skin irritation. The electrical stimulation treatment will last for 8 h per day for 2 weeks. Patients assigned to the control group will continue to receive the previous standard treatment without electrical stimulation. During treatment, all patients will receive intensive care to prevent hypotension, hypoxia, intracranial hypertension and disturbance of homeostasis.

In addition to vital signs and general data, some additional data from the case report forms (CRFs) will be assessed at the beginning of the trial and during treatment. Once enrolled in the trial, the GCS, Glasgow Coma Scale Motor Part (GMS), Full Outline of Unresponsiveness (FOUR) scale, Coma Recovery Scale-Revised (CRS-R) and Disability Rating Scale (DRS) scores of each patient will be collected.

### Study endpoint

The primary endpoint is the percentage of patients regaining consciousness 6 months after injury. The degree of consciousness can be divided into three categories: VS, MCS and consciousness (CS). The diagnosis of consciousness includes complete wakefulness and awareness of self and environment, precise comprehension and interaction, correct orientation of figures, time and location, the ability to obey commands and intact light and deep reflexes [24]. In the ACES trial, we will focus on the percentage of patients regaining consciousness 6 months after injury. This parameter indicates the long-term effect of RMNS.

The secondary endpoints are GOSE, CRS-R and DRS scores 28 days, 3 months and 6 months after injury and GCS, GMS and FOUR scores day 1 and day 7 after enrolment and 28 days, 3 months and 6 months after injury. Duration of unconsciousness and mechanical ventilation, and length of ICU and hospital stays will also be recorded as secondary endpoints.

The safety of RMNS will be assessed by the incidence of adverse events within 6 months post injury, including but not limited to (1) seizures, (2) increased intracranial pressure and (3) intracranial bleeding.

### Data collection and follow-up

General baseline information will be collected about patients when they are sent to the emergency room and will include the following: demographics, time of admission, cause of injury, whether accompanied by multiple trauma, injury severity score, initial GCS score, initial intracranial computed tomography (CT) data including Marshall score and whether complicated by subarachnoid haemorrhage, pupillary light reflex or the need of craniotomy.

After 7–14 days of standard treatment, when the patient’s condition is stable and once he/she is enrolled and randomised, additional data will be collected, including vital signs such as blood pressure, heart rate, respiratory rate and oxygen saturation, pupillary light reflex and GCS, FOUR, CRS-R and DRS scores.

Patients in both the RMNS group and the sham group will be evaluated via GCS and FOUR scores at day 1 and day 7 of the treatment. Whether or not the coma patient regains consciousness and the date will be recorded in the CRF. Any complications will also be observed and recorded during the treatment.

Outcome assessment will be performed using the GCS, FOUR, CRS-R, DRS and GOSE at 1, 3 and 6 months. Complications during follow-up will also be assessed. If the patient is still in the hospital, the investigator may visit the patient on the ward to go through the evaluation. If the patient has been discharged, his or her legal representative will be told to come back in due time for assessment after leaving the hospital. If the patient does not arrive, the investigator will try to contact the patient or the family members by telephone. Other possible methods may also be used to explain the situation and complete the outcome assessment. If all attempts fail, no further contact will be made, and the patient will be recorded as lost to follow-up. Any death occurring either in or out of the hospital will be recorded and possible causes explored.

All the data will be collected by independent investigators who are blind to the patient’s allocation. Each local study centre will assign a specific investigator at the beginning of the trial. This investigator will be excluded throughout treatment of all the participants unless asked to do the assessment by clinicians. During the 2 weeks of RMNS treatment, the investigators will be asked to collect the data during the interval of 8-h RMNS treatment per day so as to eliminate any chance of disclosing the allocation.

### Data management

All variables specified in the protocol will be documented on standardised paper CRFs in all participating centres. When the 6 months follow-up is done, data in the CRF of each patient will be validated for completeness, consistency and plausibility by an independent investigating physician in a local centre. Then the CRF will be transmitted to the coordinating centre (Shanghai Institute of Head Trauma), which will be responsible for the development of a central database and data entry and storage. At the end of the trial, the database will be locked and sent to the study statistician for analysis based on a predetermined statistical analysis plan.

### Statistics and data analysis

#### Sample size justification

The required sample size is calculated based on the results of our previous randomised controlled trial [24]. In that study, the proportions of patients regaining consciousness in the RMNS and non-stimulation groups were found to be 59.8% and 46.2%, respectively. Thus, to detect a difference of not less than 13.6% (the difference between 59.8% and 46.2%) between the experimental group and the control group, 334 patients will be needed with an α value of 0.05 and a power of 0.8. To allow for some patients lost to follow-up, a sample size of 380 in total (190 for each group) has therefore been chosen, which gives 84% power to detect the same difference at the same significance level.

#### Statistical analysis

Analysis will be performed in accordance with the intention-to-treat principle. All randomised patients will be included in their original assigned group regardless of the actual treatment approach they receive. Categorical variables will be described as numbers and percentages and analysed by the chi-squared test. Normally distributed continuous variables will be presented as mean and standard deviation (SD) and compared using Student’s *t* test; non-normally distributed variables will be expressed as median and interquartile ranges and analysed using the Mann–Whitney U test. A two-sided significance level of 0.05 will be used.

Baseline data such as age and GCS score at admission will be tested for any imbalance. If imbalances are detected, those factors will be corrected using a multiple logistic regression model.

The primary endpoint, namely the percentage of patients regaining consciousness 6 months after injury, will be presented as total percentage per group and analysed as categorical variables. The secondary outcome variables describing clinical status throughout the treatment and follow-up will be analysed as continuous variables.

## Discussion

Right median nerve electrical stimulation has been adopted as a safe, inexpensive, non-invasive therapy for the neuroresuscitation of coma patients for more than two decades. There are several advantages to stimulating the right median nerve instead of other parts of body. First, the right median nerve is a peripheral portal to the central nervous system, and the sensory representation of the hand in the cortex is disproportionately large compared to other parts of the body. Second, Broca’s motor/speech planning area is in the left frontotemporal region in most individuals. Several possible mechanisms may underlie the effects of this treatment.

The first is that the spinoreticular component of the median nerve pathway synapses with neurons of the ascending reticular activating system (ARAS) [14]. The ARAS is a complex neural network connecting the reticular formation of the brain stem to the cerebral cortex via excitatory relays in the intralaminar nuclei of the thalamus. Therefore, the ARAS plays an important role in maintaining a state of wakefulness [26–29]. Studies have shown that the ARAS is activated by RMNS applied with a painful intensity [30], which may be a pathway for the therapeutic function of electrical stimulation.

A second mechanism is related to neurotrophins such as nerve growth factor and brain-derived neurotrophic factor (BDNF). Neurotrophic factors, which play an important role in neuroplasticity, may promote synaptic remodelling and changes in receptor expression or activation [31]. Previous studies have found that BDNF might enhance the survival of neurons after a hypoglycaemic coma [32]. Studies have also shown that BDNF levels increase in environmental enrichment animals compared to those housed in standard conditions [33]. RMNS, serving as a type of environmental enrichment, may raise the concentration of neurotrophins, leading to the survival of more neurons and hastening the recovery of comatose patients.

Increases in cerebral blood flow may be another pathway through which RMNS functions. In a research project conducted by Liu and colleagues, six comatose patients underwent SPECT scans for cerebral perfusion evaluation before and after the stimulation, and brain perfusion was found to have increased in all cases [20]. Other mechanisms include RMNS-induced changes in neurotransmitters such as dopamine and glutamate [34, 35] and improved electroencephalogram activity [16].

Over the years, a great number of researchers have focused on this treatment. However, because of the limitations of the studies mentioned above, its efficacy in accelerating recovery and improving overall outcomes has not been well established. Our ACES trial addresses right median nerve electrical stimulation therapy applied at an early phase (7–14 days post injury), with a standard stimulation protocol continuing for 14 days and with follow-up for 6 months.

This trial has some novel features compared with other major RMNS trials. First, it is an Asian multicentre randomised controlled trial. The previous trials only focused on one region or even a single hospital. This trial is the first to widen the scope to a whole continent, reducing selection bias to a great extent. Second, in this trial, the RMNS therapy starts at an early stage, 7–14 days after the injury. Research shows that the later the application of electrical stimulation, the longer the duration needed to obtain a similar outcome [14]. Earlier commencement of neuroresuscitation treatment can show more efficacy, as long as the patient is stable. Third, the ACES trial uses various kinds of assessment scales to evaluate the condition of participants. The assessments fall into two groups: recovery pace parameters and long-term outcome parameters. The former group includes the GCS, GMS and FOUR. The GCS includes eye opening, verbal and motor responses. It has been used ubiquitously in acute care databases and in studies of acute neurologic injury. However, the GCS has been deficient in measuring key components of neurologic examination used for prognostication and most conspicuously lacks assessment of the brainstem reflexes. The FOUR score has been developed to overcome these inadequacies. It consists of four components that evaluate eye responses, motor responses, brainstem reflexes and respiration patterns. Because of its greater neurologic detail, the FOUR score is of more benefit than the GCS in predicting mortality in the ICU. A recent large multicentre prospective study in critically ill patients found an excellent inter-rater agreement between paired clinicians [36–38]. The long-term outcome parameters are the CRS-R, DRS and GOSE. The CRS-R is a standardised neurobehavioural assessment tool comprising six hierarchically organised subscales (auditory, visual, motor, oromotor–verbal, communication and arousal). Scores range from 0 to 23, with higher scores indicating a higher level of neurobehavioural function [1]. The DRS, a measure of functional outcome, includes measures of eye opening, verbalisation and motor response; cognitive understanding of feeding, dressing and grooming; degree of assistance and supervision required; and employability. Scores range from 0 to 29, with higher values indicating greater disability [1, 39]. The GOSE has eight ordered categories: death, vegetative state, lower severe disability, upper severe disability, lower moderate disability, upper moderate disability, lower good recovery and upper good recovery. It was developed as an extended version of the GOS in response to the perceived lack of sensitivity of the latter. It is now the primary outcome measure in trials of TBI [40]. Using all these scales, we hope to evaluate the condition of the coma patients as thoroughly as possible.

TBI-related coma remains an important topic for today’s neuroscientists owing to its high incidence, poor outcomes and the heavy burden it places on families and society. RMNS therapy is promising. It is hoped that the ACES trial will provide valuable information regarding the question: Is right median nerve electrical stimulation able to facilitate a faster awakening and a better long-term outcome in TBI comatose patients? and lead to optimal use of median nerve electrical treatment.

## Trial status

The study is currently recruiting participants.

## Additional files

Additional file 1: SPIRIT 2013 checklist: recommended items to address in a clinical trial protocol and related documents. (PDF 77 kb)

Additional file 2: Ethical approval file: ethical approval of participating centres involved. (DOCX 83 kb)

## Abbreviations

BDNF: Brain-derived neurotrophic factor
CRF: Case report form
CRS-R: Coma Recovery Scale-Revised
CS: Conscious state
DRS: Disability Rating Scale
FAM: Functional Assessment Measure
FIM: Functional Independence Measure
FOUR: Full Outline of Unresponsiveness (scale)
GCS: Glasgow Coma Scale
GMS: Glasgow Coma Scale Motor Part
GOS: Glasgow Outcome Scale
GOSE: Extended Glasgow Outcome Scale
MCS: Minimally conscious state
RMNS: Right median nerve stimulation
TBI: Traumatic brain injury
VS: Vegetative state
