# Supplementary material for: Right median nerve electrical stimulation for acute traumatic coma (the Asia Coma Electrical Stimulation trial): study protocol for a randomised controlled trial
Source: Trials. 2017 Jul 10;18:311. doi: 10.1186/s13063-017-2045-x (PMC5504835; doi:10.1186/s13063-017-2045-x)
Supplement: Supplementary file 2 — Ethical approval file: ethical approval of participating centres involved. (DOCX 83 kb) [file 13063_2017_2045_MOESM2_ESM.docx]

Ethical approval of participating centres involved

The study protocol and consent forms have been approved by

1. the Ethics Committee of Renji Hospital (NO: Renji Lunshen [2016] 001(2));
2. the Ethics Committee of Guangzhou General Hospital of Guangzhou Military Region (NO: 2016-3-001);
3. the Ethics Committee of the People’s Hospital of Shaoxin (NO: 2016 Lunshenlun 015);
4. the Ethics Committee of the 98 Hospital of the People’s Liberation Army (NO: Lunshen 2016-02);
5. the Ethics Committee of the Second People’s Hospital of Nanning (NO: [2016] 001);
6. the Ethics Committee of the Central People’s Hospital of Tengzhou (NO: 2016-020);
7. the Ethics Committee of Harrison International Peace Hospital of Hengshui (NO: 2016-2-001)
8. the Ethics Committee of the 421 Hospital of the People’s Liberation Army (NO: [2016]-002);
9. the Ethics Committee of Jiangning Hospital of Nanjing (NO: Lunshenpi 02201601);

(10) the Ethics Committee of the First People’s Hospital of Yulin (NO:

lunshen 2016-1-003);

(11) the Ethics Committee of the First People’s Hospital of Huaian (NO:

IRM-KPJ2016-003-01);

(12) the Ethics Committee of the People’s Hospital of Hunan (NO:

[2016]-01);

(13) the Ethics Committee of the People’s Hospital of Pingyang (NO:

2016-03-006);

(14) the Ethics Committee of the Second People’s Hospital of Tianshui

(NO: lunshen 2016-003);

(15) the Ethics Committee of the Fifth People’s Hospital of Zhengzhou

University (NO: 2016-001-013);

(16) the Ethics Committee of General Hospital of Beijing Military

Region (NO: 2016-046).
